# Supplementary material for: Proliferative Classification of Intracranially Injected HER2-positive Breast Cancer Cell Lines
Source: Cancers (Basel). 2020 Jul 6;12(7):1811. doi: 10.3390/cancers12071811 (PMC7408688; doi:10.3390/cancers12071811)
Supplement: Supplementary file 1 [file cancers-12-01811-s001.pdf]

cancers

Article

Proliferative Classification of Intracranially Injected HER2-positive Breast Cancer Cell Lines

Yuka Kuroiwa, Jun Nakayama, Chihiro Adachi, Takafumi Inoue, Shinya Watanabe and Kentaro Semba

Supplementary Materials

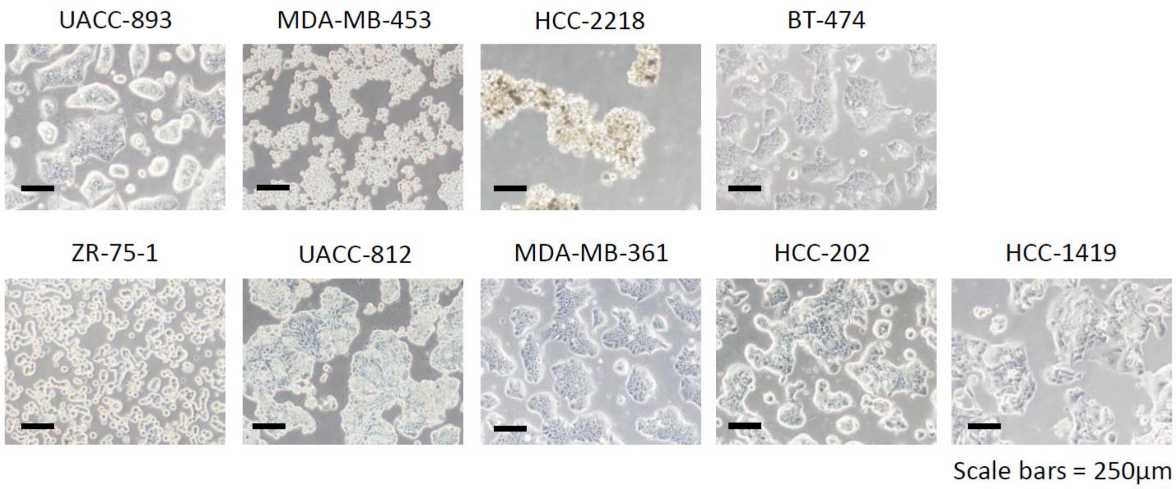

Figure S1. Cell morphology of nine HER2-positive breast cancer cell lines. Pictures of nine HER2-positive breast cancer cell lines cultured in their culture medium are shown. Scale bars = 250 μm.

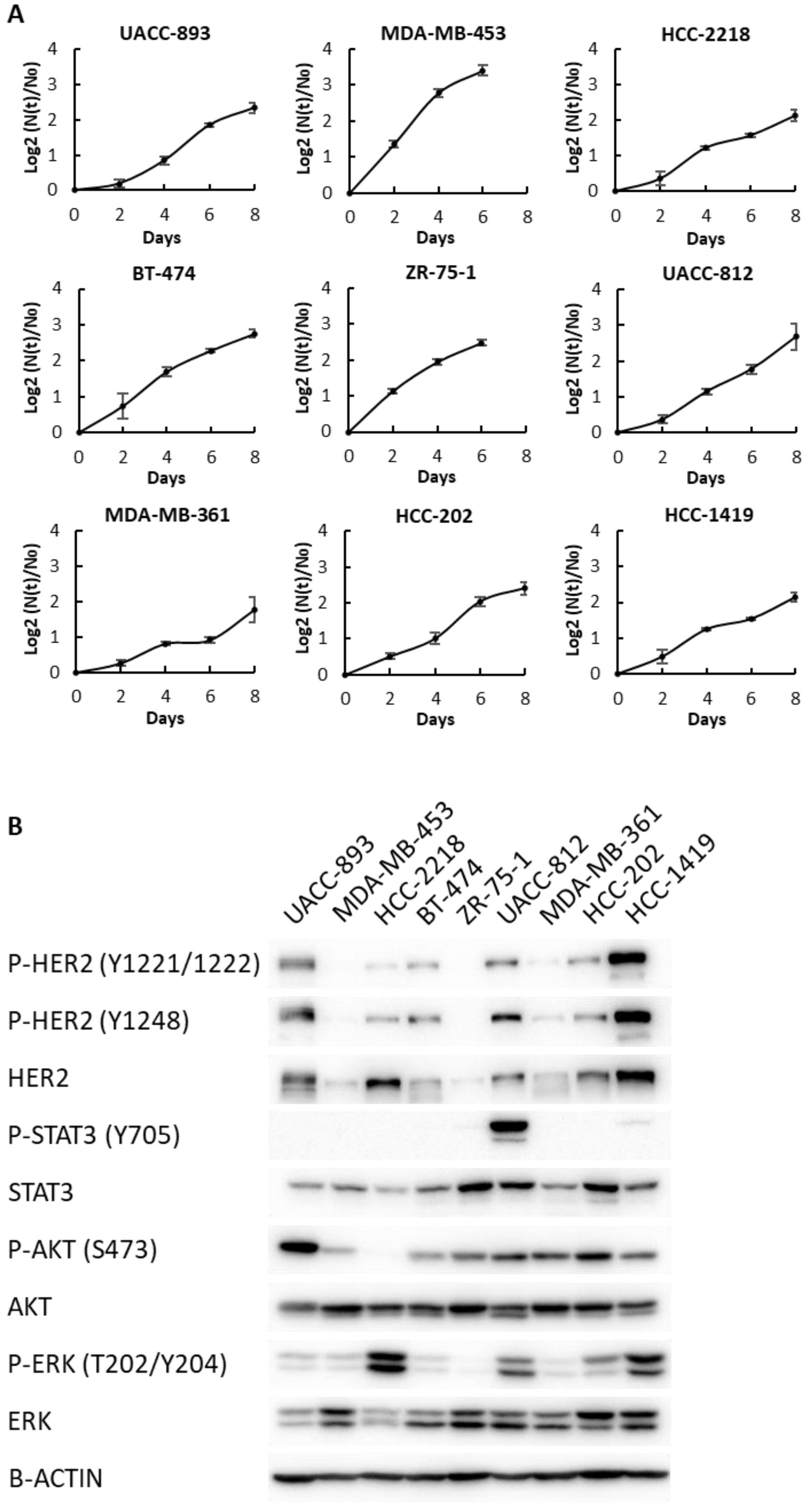

Figure S2. Proliferative activity and HER2 signaling in nine HER2-positive breast cancer cell lines. (**A**) Proliferative activity of nine HER2-positive breast cancer cell lines *in vitro*. A total of 1.5×10^5 UACC-893-luc2, MDA-MB-453-luc2, HCC-2218-luc2, BT-474-luc2, ZR-75-1-luc2, UACC-812-luc2, MDA-MB-361-luc2, HCC-202-luc2, and HCC-1419-luc2 cells were seeded in 12-well plates and incubated for 6-8 days (n=3). The cell number was counted every other day. The cell number was converted to a log2 ($N(t)/N_0$) value for each replicate and their mean value was plotted. N(t) = The cell number for each day. N_0 = The number of cells seeded on day 0 (=1.5×10^5 cells). (**B**) Western blotting of nine HER2-positive breast cancer cell lines. The cell lysates of nine luc2-introduced HER2-positive breast cancer cell lines were collected, and 15 μg of total protein was subjected to SDS-PAGE.

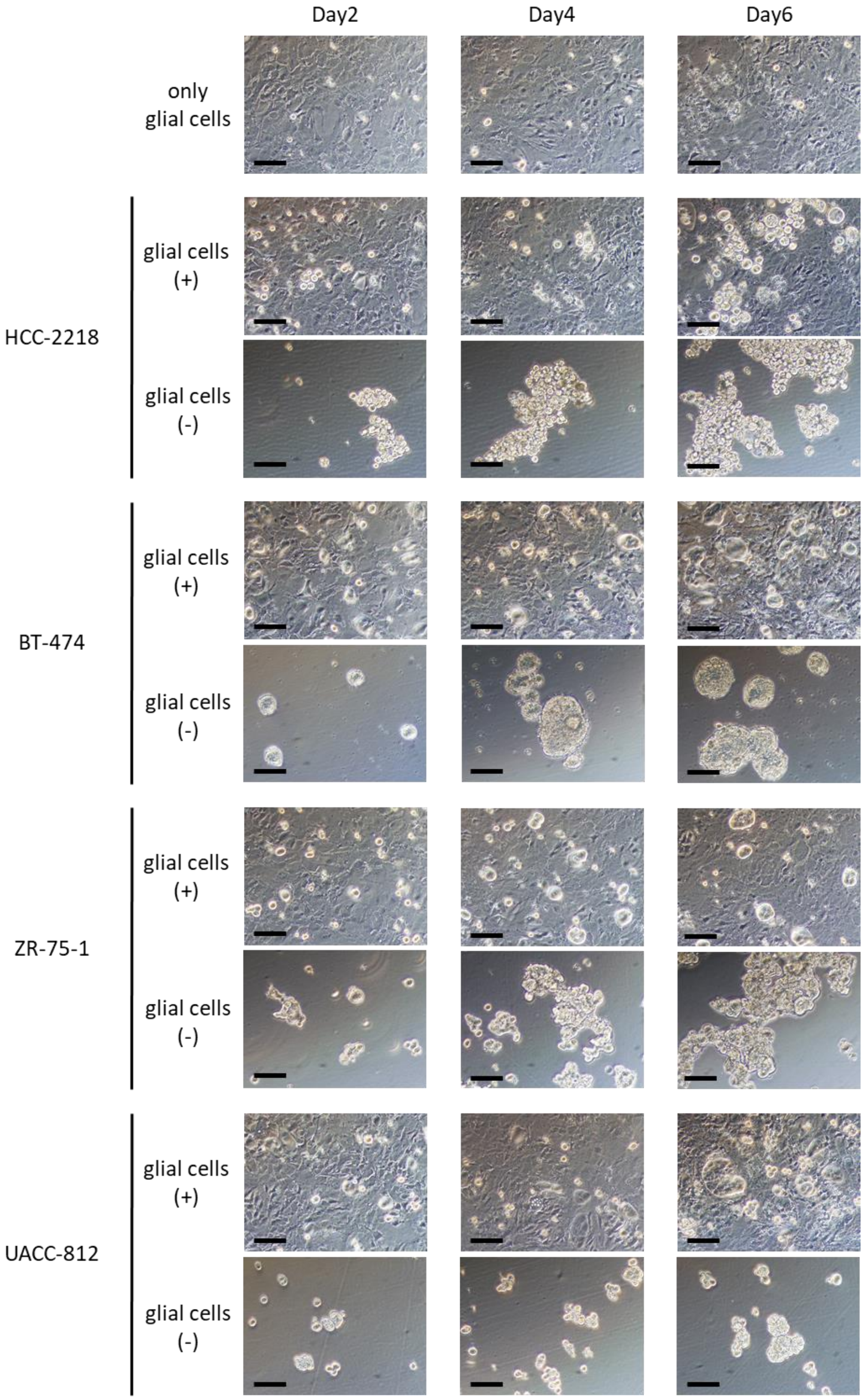
Day2
Day4
Day6
only
glial cells
HCC-2218
glial cells
(+)
glial cells
(-)
BT-474
glial cells
(+)
glial cells
(-)
ZR-75-1
glial cells
(+)
glial cells
(-)
UACC-812
glial cells
(+)
glial cells
(-)

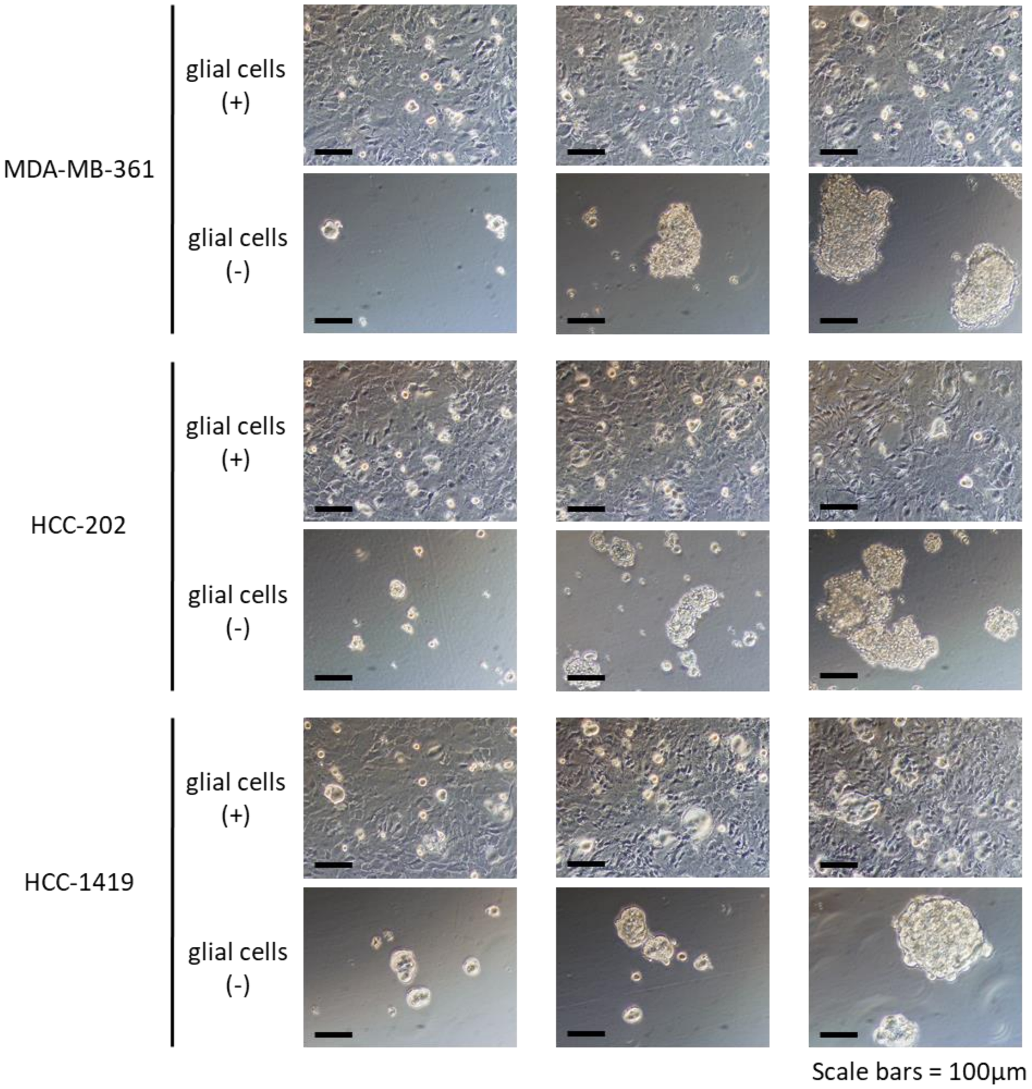

Figure S3. Coculture of MSG cell lines and mouse-derived glial cells. Pictures of seven MSG cell lines cultured in serum-free medium with or without glial cells for six days (n = 3). Pictures were taken every other day. Scale bars = 100 μm.

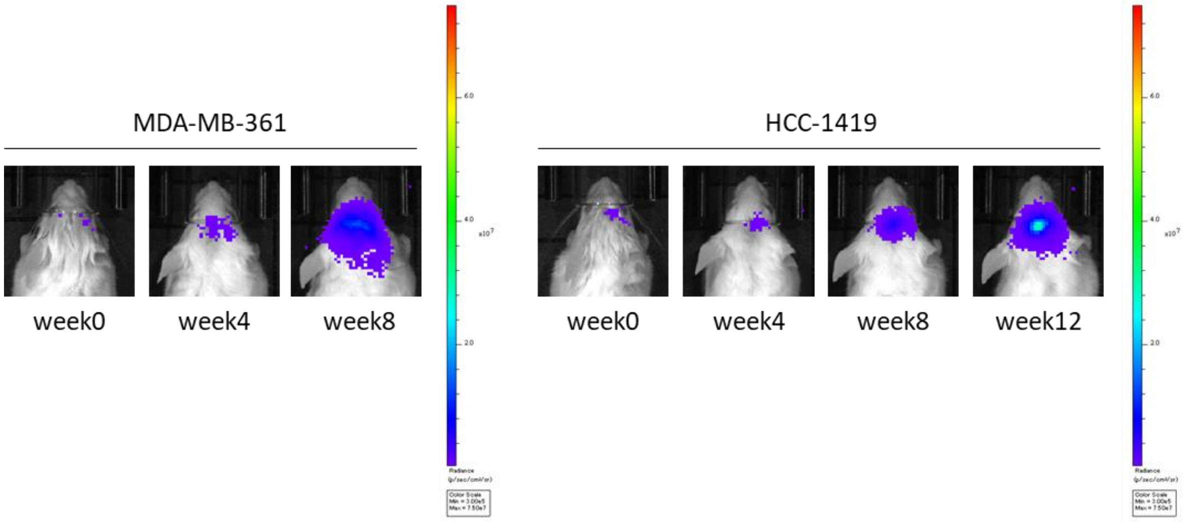

Figure S4. Intracranial injection and subsequent long-term IVIS imaging of some MSG cell lines. MDA-MB-361-luc2 and HCC-1419-luc2 cells were intracranially injected into NOD-SCID mice (MDA-MB-361-luc2, n = 3; HCC-1419-luc2, n = 4).

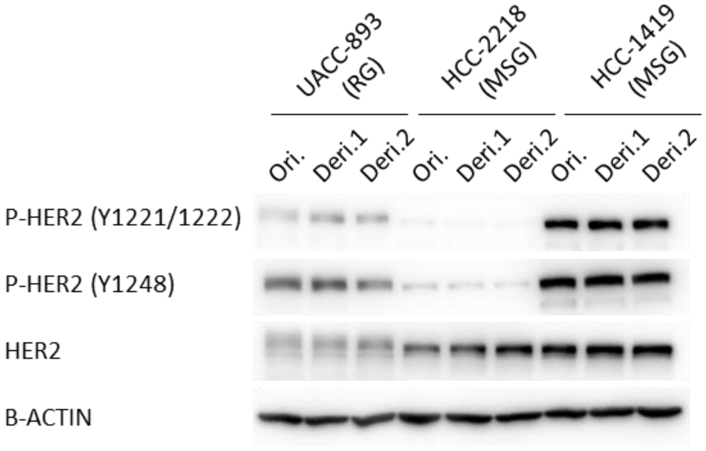

Figure S5. Western blotting of three original HER2-positive breast cancer cell lines and their derivatives that survived in the brain tissue. HER2 expression and HER2 phosphorylation were examined by western blotting. Ori.: Original luc2-introduced HER2-positive breast cancer cell lines. Deri.: Tumor cells that survived in the brain tissue. Deri.1 and Deri.2 were isolated from the brains of two different mice.

Table S1. The intensity of bioluminescence per 1 × 10^5 cells *in vitro*.

Cell line	Average bioluminescence (× 10^5 photons/sec)
UACC-893	3.400
MDA-MB-453	8.485
HCC-2218	1.469
BT-474	3.757
ZR-75-1	9.964
UACC-812	4.910
MDA-MB-361	4.043
HCC-202	1.248
HCC-1419	12.13

Table S2. Gene expression profile of nine HER2-positive breast cancer cell lines.

Cell line	ER	PR	HER2	EGFR	TP53	PTEN	References
UACC-893	-	-	+	+/-	-	+/-	[29,34,36–38,42,45,46]
MDA-MB-453	-	-	+/-	-	-	+	[34,36–40,42,45,46,48]
HCC-2218	-	-	+	-	+	+	[31,37,38,46]
BT-474	+/-	+	+	-	+	+	[32,34,36–39,42,45,46,48]
ZR-75-1	+/-	+/-	+/-	-	-	+/-	[33,34,36,37,39,41,42,45,46,48]
UACC-812	+/-	+/-	+	-	-	+	[29,34,36–38,45,46,48]
MDA-MB-361	+	+/-	+	-	-	+	[34,36–38,42,45,46,48]
HCC-202	-	-	+	+/-	-	+	[31,36–38,46,48]
HCC-1419	+/-	-	+	-	-	+	[31,36–38,46]

+/- : There are conflicting reports on gene expression profile. ER: Estrogen receptor, PR: Progesterone receptor, HER2: Human epidermal growth factor receptor type2, EGFR: epidermal growth factor receptor, TP53: tumor protein p53, PTEN: phosphatase and tensin homolog. In this study, cell lines with HER2 expression were all considered as "HER2-positive" regardless of ER/PR status, since subtyping of breast cancer cell lines is not unified in previous studies [49].

Table S3. Genes that are mutated in the RG but not in the MSG.

Gene			Mutation			
			UACC-893		MDA-MB-453	
**Official gene symbol**	**Gene name**	**Loci**	Variant type	Protein change	Variant type	Protein change
BAZ2B	bromodomain adjacent to zinc finger domain 2B	2q24.2	SNP	D1449H	SNP	S2019C
CEP152	centrosomal protein 152	15q21.1	SNP	K1202Q	SNP	Q84E
CERS3	ceramide synthase 3	15q26.3	SNP	L290F	SNP	P280T
IRAK1	interleukin 1 receptor associated kinase 1	Xq28	SNP	P162S	SNP	S568L
LTN1 ¶	listerin E3 ubiquitin protein ligase 1	21q21.3	SNP	I600V	SNP	D695N
			Frame shift insertion	A1733fs	Frame shift insertion	A1733fs
MDN1	midasin AAA ATPase 1	6q15	SNP	S3479*/ W2538R	Frame shift deletion	AAL1404fs
MIDEAS	mitotic deacetylase associated SANT domain protein	14q24.3	SNP	D537N	SNP	E424Q
PHF3	PHD finger protein 3	6q12	SNP	E376*	SNP	P1881L
PIK3CD	phosphatidylinositol-4,5-bisphosphate 3-kinase catalytic subunit delta	1p36.22	SNP	R821C	SNP	G4E
SART3	spliceosome associated factor 3, U4/U6 recycling protein	12q23.3	SNP	S10L	SNP	S797F
SERPINI2	serpin family I member 2	3q26.1	SNP	E170*	SNP	E115K
SLC28A2	solute carrier family 28 member 2	15q21.1	SNP	T147R	SNP	R615T
TEAD3	TEA domain transcription factor 3	6p21.31	SNP	E204Q	SNP	T335I
WWC1 §	WW and C2 domain containing 1	5q34	In frame deletion	G866del	SNP	E353Q
ZNF711	zinc finger protein 711	Xq21.1	SNP	Q705E	SNP	D304V

Mutation data in Table S3 was obtained from CCLE database. Mutations without protein change and mutations in splice sites were not regarded as gene mutations in this study. ¶ A mutation without protein change was observed in HCC-202 cells. § A mutation without protein change was observed in MDA-MB-361 cells.
